# Investigation of laboratory confirmed Dengue outbreak in North-eastern Kenya, 2011

**DOI:** 10.1371/journal.pone.0198556

**Published:** 2018-06-07

**Authors:** Mark Obonyo, Ahmed Fidhow, Victor Ofula

**Affiliations:** 1 Kenya Field Epidemiology and Laboratory Training Program, Ministry of Public Health and Sanitation, Nairobi, Kenya; 2 Arbovirology/Viral Hemorrhagic Fever Laboratory, Centre for Virus Research, Kenya Medical Research Institute, Nairobi, Kenya; Public Health England, UNITED KINGDOM

## Abstract

The first laboratory confirmed dengue outbreak in Kenya was reported in coastal towns of Malindi and Kilifi in 1982. Since then, no other outbreak had been confirmed in Kenya. Dengue outbreak was confirmed among African Mission soldiers in Somalia (AMISOM) between May to October 2011. From September 2011, an upsurge of febrile patients who were negative for malaria on microscopy were reported in several health facilities in Mandera town, an adjacent area to Somalia in northern Kenya. We investigated a suspected dengue outbreak in Mandera town from 26^th^ September 2011 to 5^th^ October 2011. A suspected case was defined as acute onset of fever with two or more of the following: headache, arthralgia, myalgia, rash and hemorrhages and negative malaria microscopy results in a person presenting to a health facility in Mandera town from 1^st^ August to 2^nd^ October 2011. We prospectively identified new cases meeting the suspect case definition from 2^nd^ October to 5^th^ October 2011 and we collected blood samples from consenting patients. Blood was collected into plastic vacutainers and stored in dry shipper at -80^o^c to laboratory for dengue virus testing using real time reverse transcriptase polymerase chain reaction (rRT-PCR). We administered a standardized form to obtain clinical information. We calculated descriptive statistics to describe the outbreak. A total of 1,332 patients had been line listed by the district surveillance team, of which 381 (29%) met our suspect case definition of dengue. Cases peaked between 10^th^ September and 1^st^ October 2011 and thereafter declined. We prospectively identified 33 cases meeting the suspect case definition, of whom 30 (91%) were positive for dengue virus serotype 3 by PCR. Among the 30 laboratory confirmed patients, 20 (67%) required hospitalization (Median hospitalization period, two days with a range: 1–4 days)). And of these, 26 (86%) patients reported aches and pain, 16 (53%) reported vomiting, and four (13%) gingival bleeding. Twenty-three (77%) received anti-malarial therapy. Among laboratory-confirmed dengue patients, seven (23%) had malaria co-infection. This was the second confirmed Dengue outbreak in Kenya, and highlighted the need for improved surveillance to better define disease burden and continuous education to medical personnel to improve detection and clinical management. We also recommended enhanced community education for disease prevention.

## Introduction

Dengue the most widespread mosquito-borne viral disease results in approximately 500,000 hospitalizations and over 50,000 deaths annually, mainly among children [1,2]. Case fatality rates exceeding 5% have been reported among untreated cases of severe dengue [3]. A global study on dengue prevalence, estimates that approximately 3.9 billion people in 128 countries are at risk of infection with dengue virus (DENV) [4]. Yet In another study, it is estimated that there are 390 million cases of dengue reported annually worldwide of which approximately 96 million manifest clinically as dengue or severe dengue. Among those that clinically manifest, 14% (16 million) are reported in Africa [5]. This is significantly larger burden than previously estimated 0.1–1 million cases annually [6].

In Africa, dengue outbreaks have been reported for several decades [6,7]. In Eastern Africa, a systematic review documenting dengue outbreaks between 1823 and 2015 highlights the public health importance of dengue outbreaks in eastern Africa, its burden, associated risk factors, its predictability and investigations strategies adopted during the outbreaks. The systematic review highlights that understanding epidemiology of dengue and clinical symptoms are important in preventing future outbreaks [8]. This report describes dengue outbreak investigations in northeastern Kenya in September 2011.

An outbreak of dengue was confirmed in Somalia which borders Mandera town (located in Mandera east district) among African union mission soldiers (AMISOM) serving in Somalia between May and October 2011 in which DENV type 1, 2 and 3 were detected in over 100 soldiers [8,9]. In September, 2011 the Kenyan Ministry of Health (MoH) received reports from the Mandera district disease surveillance office in northeastern part of Kenya, regarding an unusually high number of patients presenting to several health facilities (both private and public) with fever and who were negative for malaria test on microscopy. On instructions from the MoH in conjunction with the viral hemorrhagic fever (VHF) laboratory at the Kenya Medical Research Institute (KEMRI), the district surveillance officer collected 11 blood samples from some of the febrile patients who were malaria test negative and submitted the samples to VHF laboratory for arbovirus testing. Six of the samples were positive for dengue virus type 3 on real time reverse-transcriptase-polymerase-chain-reaction (rRT-PCR) (Table 1). We conducted an investigation in Mandera east district from 26^th^ September 2011 to 5^th^ October 2011 to describe the epidemiological and clinical features of the outbreak.

**Table 1:** Results from the initial patients tested for dengue and other arboviruses using RT-PCR, September 2011.

Patient Id	Age	Sex	Date of sample collection	RT-PCR Results
1	25	Female	21/09/2011	Negative
2	19	Male	21/09/2011	Positive
3	40	Female	20/09/2011	Positive
4	18	Male	21/09/2011	Negative
5	17	Male	20/09/2011	Negative
6	35	Male	21/09/2011	Negative
7	24	Female	21/09/2011	Positive
8	23	Male	21/09/2011	Negative
9	18	Male	21/09/2011	Positive
10	23	Female	21/09/2011	Positive
11	54	Male	21/09/2011	Positive

## Materials and methods

### Site

The outbreak was reported from Mandera town located in Mandera east district in northeastern Kenya which borders Ethiopia and Somalia with a population of 288,687 persons according to 2009 Population Census. Mandera east district has two rain seasons (April to May and October to December) with annual rainfall of 225 mm. Daily temperatures are typically above 30°C (86°F), while at night, they can fall to 20°C (68°F). There is free movement of people between Shuftu (Ethiopia), Bulla Hawa (Somalia) and Mandera town (Kenya) because of porous borders.

### Characterizing the outbreak

We reviewed surveillance data prepared by the district surveillance officer (DSO) of patients with suspected dengue from 1^st^ August to 1^st^ October 2011. The case definition adopted by the DSO was any febrile patient attending a health facility in Mandera town with or without a malaria test results on microscopy from 12 health facilities (one public hospital, three private hospitals and eight private clinics). The DSO captured the patients’ details using a standardized surveillance form developed by the Kenya MoH. We re-defined a suspected case as fever (or history of fever) of acute onset in a patient of any age residing in Mandera town and presenting to any of the reporting health facilities with a negative microscopy result for malaria on blood smear test. Patients reported to have fever but had no records of malaria laboratory testing were excluded from analysis.

### Prospective case finding

Since limited laboratory testing was performed on the initially identified suspect dengue cases, we conducted facility-based prospective case finding at five facilities that had reported the highest number of suspected dengue cases, which included the district government hospital, two private hospitals and two private clinics. A suspected case was defined as person presenting from 2^nd^ to 10^th^ October 2011 with fever ≥38°C and ≥2 of the following signs or symptoms: headache, retro-orbital eye pain, arthralgia, myalgia, rash or hemorrhagic tendencies. We administered a standardized questionnaire to patients meeting the suspect case definition and we collected clinical, hospitalization, treatment, final outcome as well as awareness on dengue and exposure information. We collected blood samples from consenting patients and transported to the VHF laboratory in Nairobi, where specimens were tested for dengue, Chikungunya, yellow fever, Rift Valley fever, and West Nile viru**s** infections using rRT-PCR.

### Laboratory methods

A laboratory confirmed case was defined as any suspected case with a blood specimen positive for dengue virus by rRT-PCR. Blood specimens were centrifuged at 2000 rpm for five minutes to obtain serum. Viral RNA was then extracted from the serum using the QIAamp Viral RNA Mini Kit (Qiagen®, Maryland, USA). The RNA was tested for dengue virus by real time RT-PCR using CDC provided multiplex dengue virus type specific dengue primers and the Agpath-ID™ one step RT-PCR kit (Life Technologies, New York, USA) to differentiate various dengue sero-types. Appropriate negative and positive controls were tested alongside the test samples. The thermo-cycling conditions of the rRT-PCR run was set at reverse transcription at 45°C for 10 minutes, Taq polymerase denaturation at 95°C for 10 minutes, followed by 45 cycles of denaturation at 95°C for 15 seconds and annealing/extension at 55°C for 1 minute. Fluorescence was read at the annealing/extension step and was recorded as cycle threshold (CT) value. A run was considered successful if both the controls worked as expected. Specimens were considered positive if the CT was <40, indeterminate if the CT ≥ 40 and negative if the CT value was undetermined [10,11]. Similar laboratory procedure was used for testing the blood specimens for other arboviruses (Chikungunya, yellow fever, Rift Valley fever, and West Nile virus) using viral specific CDC provided primers and appropriate negative and positive controls.

### Data management

We recorded the data using Microsoft excel 2007 (Microsoft, Seattle, WA, USA), and analyzed using EPI Info version 3.5.3 (CDC, Atlanta, GA, USA) and Microsoft Excel 2007. We calculated descriptive statistics for all relevant variables where we calculated proportions for categorical variables and means and medians for continuous variables to describe the epidemiological and clinical characteristics of the outbreak.

### Human subjects protection

This investigation was considered a public health response to an acute event by the Kenyan Ministry of Health (MOH), and as such, did not require review by an institutional review board. The investigational protocol was approved by the Kenyan MOH. During the interviews informed consent was not obtained from the patients, as these interviews were conducted strictly as a public health response activity. However blood samples were only collected from consenting patients. Measures were taken to assure confidentiality of the information provided during these interviews. Review of surveillance data was conducted as part of routine surveillance by the MOH, and did not include any personal identifying information. Measures were taken to assure collected data were properly stored and secured.

## Results

### Characterizing the outbreak

**Fig 1:**
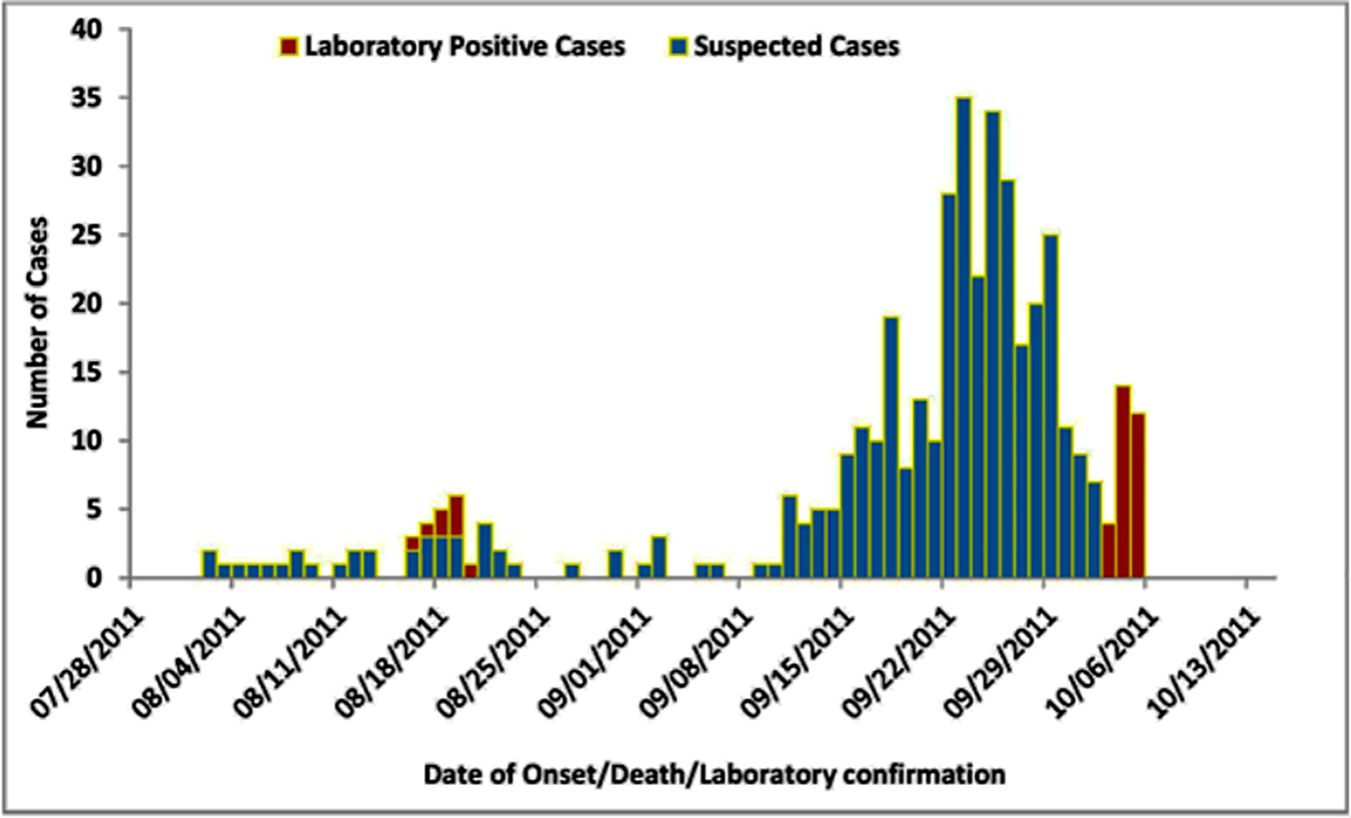
Distribution of suspected and confirmed dengue cases by date of onset and date of confirmation, Mandera east district, 2011.

A total of 1,332 febrile illness patients in 12 health facilities in Mandera were reported from 1^st^ August to 3^rd^ October 2011. Among these, 174 (13%) had positive malaria microscopy and 777 (58%) did not have any record of malaria microscopy performed, resulting in 381 (29%) case-patients who met the suspected dengue case definition. Among suspected dengue case-patients, the median age of 331 (87%) whose ages were recorded was 20 years (range: <1–82 years); there were 225 (59%) males and 115 (33%) were children <10 years old (Table 2). Weekly case reporting started increasing from 4^th^ to 10^th^ September, 2011 and remarkably increased from 11^th^ to 17^th^ September, 2011, peaking during 18^th^ to 24^th^ September, 2011. Case reporting by the health facilities remained high until 30^th^ September 2011, after which there was decline (Fig 1). All suspected cases came from villages within Mandera town with eight cases coming from neighboring villages in Ethiopia (Bulla shuftu) and Somalia (Bulla hawa).

**Table 2:** Distribution of suspected dengue fever cases by age group during the outbreak in Mandera east district, 2011.

Age group	Sex	Reported cases (n = 353)*	Percentage (%)
<10 years	Male	73	(35)
	Female	42	(29)
10–19 years	Male	51	(24)
	Female	34	(24)
20–29 years	Male	45	(22)
	Female	38	(26)
>30 years	Male	40	(19)
	Female	30	(21)

*28 case-patients had their ages missing

### Prospective case finding

We prospectively identified 33 newly presenting suspected cases of dengue from whom we collected blood specimens and tested for dengue infection. Out of these, 30 (90%) were positive for dengue virus type 3 and negative for all other dengue sub-types and other arboviruses. Among the confirmed cases, 29 (97%) had headache and joint pains, 25 (83%) muscle pains, 22 (73%) abdominal pain, 17 (57%) dizziness, 16 (53%) vomiting, four (13%) hemorrhages and two (7%) skin rash. The median number of days before seeking care from a healthcare professional after the onset of fever was two days (range: 0–7 days). Twenty (67%) patients with confirmed dengue required hospitalization. The median number of days of hospitalization among the confirmed patients was two days (range: 1–4 days). Three (75%) patients with hemorrhagic manifestations were hospitalized each for two days. Seven (23%) confirmed dengue patients were also malaria positive on thin blood smear test. All the patients with dual infection had fever, headache and joint pain; five (71%) had vomiting and dizziness; four (57%) muscle and abdominal pains; one (14%) jaundice and hemorrhagic manifestations. The median hospitalization for patients’ with dual infection was 1 day (range: 1–4 days). Among the confirmed patients, twenty-four (80%) were treated with antipyretics, 23 (77%) with anti-malarial, 15 (50%) with re-hydration fluids and 14 (47%) with antimicrobials (Table 3). None of the patients died and none required further hospitalization.

**Table 3:** Distribution of signs and symptoms among laboratory confirmed dengue fever cases in Mandera east district, 2011.

Clinical characteristics	Frequency (n = 30)	Percentage (%)
Fever	30	(100)
Headache	29	(97)
Arthralgia	29	(97)
Myalgia	25	(83)
Abdominal pain	22	(73)
Dizziness	17	(57)
Vomiting	16	(53)
Hemorrhages	4	(13)
Skin Rash	2	(7)
Jaundice	1	(3)
Hospitalized	20	(67)
Test positive for malaria*	7	(23)
Dengue and malaria co-infection	4	(57)
Treated with anti-malarial drugs	23	(77)
Treated with anti-pyretic drugs	23	(77)
Treated with re-hydration fluids	15	(50)
Treated with anti-microbial drugs	14	(47)

*Test positive for malaria on thin and thick blood smear test

A total of 29 (88%) knew of a disease outbreak in Mandera east district, 15 (46%) were made aware through a local vernacular radio station. A total of 24 (73%) had heard or seen someone else with similar clinical presentation as themselves in the recent past, of which 11 (54%) were from a member of their household. Only, 12 (36%) knew how dengue is transmitted but 31 (94%) reported that they had noticed an increase in mosquito population and similar number reported that they had been bitten by a mosquito during the day. None of the respondents used any form of protection from mosquito bites during the day and only three (9%) reported having received indoor and outdoor residual spraying by health authorities. Heaping of garbage within the compound (n = 15 (46%)) was the commonest mode of garbage disposal by the respondents and 17 (52%) self-reported presence of bushes around their residence of which 15 (88%) were less than 50 meters away from their houses (Table 4).

**Table 4:** Awareness of dengue and distribution of exposure factors among suspected and confirmed dengue cases in Mandera east, 2011.

Variable	Frequency (n = 33)	Percentage (%)
Heard of disease outbreak in Mandera East district recently	29	(88)
Source of information		
Local FM stations	15	(46)
Neighbor	12	(36)
Family member or friend	9	(27)
Religious leaders	8	(24)
Seen or heard anyone with similar illness to self	24	(73)
Household member	11	(54)
Neighbor	13	(46)
Know that disease is transmitted by mosquito bites	12	(36)
Noticed increase in mosquito population in recent past	31	(94)
Bitten by mosquitoes in past 7 days during the day	31	(94)
Place of sleep during the day		
Inside the house	32	(97)
Outside the house	1	(3)
Always use a net while sleeping during the day	2	(9)
Use insect repellants to protect self	0	(100)
Use protective clothing (long sleeves shirts, dresses and shorts to protect self	0	(100)
Have had indoor residual spraying and outdoor residual spraying	3	(9)
Types of water storage facility		
Underground water tanks	18	(55)
Overhead water tanks	3	(9)
Jerry cans	12	(36)
Duration water stays in water storage tanks		
More than a week	10	(48)
Less than a week	11	(52)
Self-reported presence of garbage around place of residence	14	(42)
Methods of garbage disposal		
Dug pits	7	(21)
Garbage bins	14	(42)
Heaped garbage	15	(46)
Self-reported presence of bushes around place of residence	17	(52)
Distance of bushes around place of residence		
Less than 50 meters	15	(88)
More than 50 meters	2	(12)

## Discussion

We report on the laboratory-confirmed dengue outbreak caused by dengue virus type 3, in northeastern Kenya which is believed to have spread from Somalia. Prior to this outbreak, the only documented and laboratory confirmed dengue outbreak in Kenya was reported in the coastal towns of Malindi and Kilifi in 1982 [12]. Since then, evidence of circulation of dengue virus (DENV) has been demonstrated in various sero-prevalence and sero-incidence surveys conducted in coastal and inland parts of Kenya [13–15]. This outbreak in Mandera was predominantly caused by DENV subtype 3, whereas the previously reported outbreak in 1982 in Malindi and Kilifi was reported to be caused predominantly by DENV subtype type 2 and was believed to have spread from the Seychelles outbreak of 1979 [16]. Cases of dengue continued to be reported in Mandera east district for the remainder part of 2011 and blood specimens that were later tested confirmed three DENV subtypes (DENV 1, 2 and 3) [17]. Since this outbreak in Mandera east district, other outbreaks of dengue have been confirmed in Kenya, specifically Mombasa County between February and March 2013 in which 58% (155/267) suspected dengue cases were confirmed to have DENV infection with DENV serotypes 1, 2 and 3 confirmed with laboratory testing [18]. A subsequent community survey in Mombasa County in 2013 among 1,500 participants from 701 households, showed that 210 (13%) had evidence of recent DENV infection [19]. Subsequently in 2013, a study utilizing 1,091 HIV-negative blood specimens from the 2007 Kenya AIDS Indicator Survey (KAIS 2007) were tested for the presence of IgG antibodies to DENV, chikungunya virus (CHIKV) and Rift valley fever virus (RVFV). DENV antibodies were detected in all previous administrative provinces of Kenya except in Nairobi [15]. In January 2017, outbreaks of dengue were reported in Mombasa and Wajir Counties affecting more than 1000 people with more than 500 having been laboratory confirmed to have DENV infection [20]. Although there is limited surveillance for dengue in most Eastern African countries, outbreaks of suspected dengue have been reported in Eastern Africa since 1960’s [6]. The Seychelles reported an outbreak between 1977 and 1979 affecting more than 75% of a population [4,6,16,21]. The islands of Comoros, in the Indian Ocean, experienced an epidemic in 1993 that affected more than 56, 000 persons with an estimated attack rate of nearly 30% [22]. More recently, Dengue outbreak affecting 100 people occurred in Tanzania in 2014 and was caused by DENV sero-type 2 [23]. A large outbreak of dengue resulting in more than 17,000 cases was documented in the Cape Verde islands in 2009 [6,7,21,24]. A detailed description of Dengue outbreaks that have occurred in Eastern Africa is documented in a systematic review published in 2016 [8]. These outbreaks of DENV infections and various surveys that have demonstrated circulation of DENV are an indication that DENV is likely endemic in several parts of Eastern Africa where the mosquito vector is widely present and more outbreaks are likely to continue to be reported in Kenya and other countries in Eastern Africa.

During this dengue outbreak Mandera east district, most of the patients with laboratory confirmed dengue were treated for malaria despite laboratory testing showing they were negative for malaria. In Kenya and most of Africa where malaria, typhoid and other febrile diseases are endemic, over 70% of febrile illnesses are treated as presumptive malaria or typhoid, often without proper medical examination and a laboratory diagnosis [6]. Many patients in Africa with fever are designated as having fever of unknown origin or malaria and remain without a diagnosis even if they fail to respond to anti-malarial drugs [25]. Under these prevailing practices, there is a potential for misdiagnosing dengue as malaria or other endemic fevers, such as typhoid and leptospirosis [6,25]. These problems have implications for clinical management of dengue, as well as for implementation of adequate surveillance for the disease, and highlight the need to establish a robust surveillance system for dengue. However in most countries in Africa, funding for surveillance and other research activities pertaining to dengue has been limited, this mainly owing to the assumption that dengue is not a significant health problem in the region [6]. The International Health regulation (IHR), in Africa implemented under Integrated Disease Surveillance and Response (IDSR) is an ideal entry point to address the poor surveillance of dengue and other diseases. If full compliance is achieved by African countries, there will be tremendous improvement in dengue surveillance as well as other diseases.

Some of the possible exposure factors which might have fueled the outbreak included: use of underground water tank as primary mode of water storage; inconsistent use of nets during the day while sleeping; lack of use of any protective method to prevent mosquito bites and presence of bushes around the places of residence less than 50 meters from the house. This in addition to the environmental factors like temperature and rainfall patterns have been identified as possible risk factors for DENV infection [18,26–28]. However due to small sample size from prospectively identified suspected dengue cases and subsequently large numbers that turned positive for DENV infection on laboratory testing, we were unable to evaluate statistically association between DENV infection with the exposure factors identified. Some of the public health actions undertaken to control the outbreak included advocacy, communication and social mobilization of the residents of Mandera town mainly through religious leaders and local FM radio stations mainly to reduce anxiety, clear speculations and myths and encourage participation in vector control activities through clearing of garbage. There was also sensitization of all medical personnel within Mandera town on dengue detection and case management and vector control exercises, indoor residual spraying (IRS) of 16,000 households including government prison and boarding schools; outdoor residual spraying (ORS) through fogging in villages where most cases were reported and spraying of underground water tanks with larvicidals.

Our findings are subject to several limitations. First, case reporting was conducted by healthcare workers with other clinical responsibilities likely leading to under reporting and limited quality of data captured. This was evident by incompleteness of information collected for suspect cases, such as age of the patient. More so, the surveillance form used to capture patient information lacked space for entry of clinical information. Second, lack of a proper surveillance system for dengue coupled by poor diagnostic capabilities made it difficult to test a larger number of suspect cases for dengue, limiting our capacity to more comprehensively describe the outbreak and further characterize the confirmed dengue patients.

This dengue outbreak of 2011 in Mandera Kenya was the second laboratory confirmed dengue outbreak in nearly three decades. The outbreak highlighted the need for improved surveillance of acute febrile illness in the region to better define disease burden, seasonality and trends. Although education on prevention practices like wearing protective clothing, such as long sleeve shirts and long pants, environmental management, improved sanitation, proper disposal of plastics and other open containers and use of insect repellants is important for minimizing morbidity and mortality due to dengue, the real message should be on proper case management which is important for minimizing severe outcome due to dengue virus infection. Sustained, systematic surveillance for dengue-like illness combined with improved laboratory diagnostics and education of health care providers on proper case management of dengue, is critical in future prevention and control strategies for dengue in Kenya and indeed Africa. There is also need for a well-structured epidemiological study preferably a community based survey to determine the true burden of dengue in the region and vector assessments for specific vector recommendations in Mandera East district and surrounding regions.
